# The relationship between lipid profile and severity of liver damage in cirrhotic patients

**Published:** 2010-12-01

**Authors:** Mohammad Reza Ghadir, Ali Akbar Riahin, Abbas Havaspour, Mehrdad Nooranipour, Abbas Ali Habibinejad

**Affiliations:** 1 Department of Gastroenterology,Qom University of Medical Sciences, Qom, IR Iran; 2 Department of Infectious Diseases,Islamic Azad University of Qom, Qom, IR Iran; 3 Islamic Azad University of Qom, Qom, IR Iran; 4 Baqiyatallah Research Center for Gastroenterology and Liver Disease, Tehran, IR Iran

**Keywords:** Cirrhosis, Lipid profile, MELD score

## Abstract

**Background and Aims:**

An impaired lipid metabolism is often observed in patients with chronic liver diseases. To determine lipid profile in patients with cirrhosis and to asses if it relates to the severity of the cirrhosis.

**Materials and Methods:**

In an analytical cross-sectional study, 50 patients with cirrhosis (case) and 50 age- and sex matched healthy normolipidemic patients (comparison) were studied. A questionnaire including personal characteristics,etiology of cirrhosis, pathologic criteria of CHILD and MELD and lipid profile (total, LDL, and HDL cholesterol and triglyceride) was completed for each patient.

**Results:**

In patients with cirrhosis, there was a significant decrease in serum triglyceride, total, LDL and HDL cholesterol levels compared to the comparison group (mean of 82 vs 187, 138 vs 184, 80 vs 137, and 40 vs 44 mg/dL, respectively; all p<0.05). Comparison of lipid profile with pathologic progression of cirrhosis revealed that except for serum triglyceride level, serum lipid levels diminishe linearly with progression of liver damage.

**Conclusions:**

Serum total, LDL and HDL cholesterol level in patients with cirrhosis is inversely correlate with severity of cirrhosis.

## Introduction

Lipids are one of the necessary components which control cellular functions and homeostasis. Liver plays an essential role in lipid metabolism, several stages of lipid synthesis and transportation. Therefore, it is reasonable to expect an abnormal lipid profile in those with sever liver dysfunction. There is prominent decline in plasma cholesterol and triglyceride (TG) levels in patients with severe hepatitis and hepatic failure because of reduction of lipoprotein biosynthesis. For reduced liver biosynthesis capacity, low levels of TG and cholesterol is usually observed in chronic liver diseases [1].

Major complications of cirrhosis include portal hypertension, esophageal varices, hepatomegaly, hypersplenism, ascites, spontaneous bacterial peritonitis, hepatorenal syndrome type I and II,  hepatic encephalopathy, hepatopulmonary syndrome, malnutrition, coagulapathy, fibrinolysis factor deficiency, thrombocytopenia, bone disorders, osteopenia, osteoporosis, osteomalacia, hematologic disorders, anemia, hemolysis, neutropenia, diabetes mellitus, cancer, heart or renal failure, pancreatitis, etc [2][3][4]. Cirrhotic patients need frequent visits and multiple hospitalizations for management of cirrhosis or its complications. However, choosing the proper treatment plan depends on the severity, type of liver damage and possibility of assessing its extent. To evaluate cirrhosis, Child-Turcotte-Pough criteria can be used [2]. In addition, MELD criteria are used to choose liver transplantation candidates which are substituted by PELD criteria for children under 12 years [5]. AS mentioned earlier, metabolism of TG, cholesterol and synthesis of lipoproteins predominantly occurs in liver and various parenchymal diseases may lead to alterations in lipoproteins structure and transfer through the blood [6]. Although, several studies have been performed on lipoprotein profile alterations in those with liver disease, contributions from Iran are scarce. Despite diverse results, some outcomes have been observed to be common [7][8][9][10][11][12][13][14][15].Due to the high prevalence of chronic liver disease in our country, we conducted this study to determine lipid profile in patients with cirrhosis and to assess if it relates to the severity of cirrhosis.

## Materials And Methods

In an analytical cross-sectional study, out of 212 cirrhotic patients admitted between 2005 and 2008 to Kamkar Hospital, a referral center in Qom, Central Iran, 50 patients were selected to enroll into our study, after excluding those with diabetes mellitus, cancer, renal failure, acute pancreatitis, and acute gastrointestinal bleeding, and patients with history of hyperlipidemia, recent parenteral nutrition, history of taking glucose or lipid lowering drugs. A questionnaire including personal characteristics such as age, gender and etiology of cirrhosis (e.g., HBV, HCV, drugs and toxins, chronic liver congestion, Wilson's disease, autoimmune hepatitis, hemochromatosis, α1-antitripsin deficiency and cryptogenic cirrhosis) was completed for each patient. Then the diagnostic method used for the diagnosis of cirrhosis was determined. The methods included either liver biopsy or combination of clinical signs and symptoms and sonography. The next part of the questionnaire included presence of ascites and/or encephalitis grading in a 3-point scale according to Child criteria. Serum TG level, total, HDL and LDL cholesterol were then measured. Finally, Child-Turcotte-Pough and MELD criteria were calculated for each patient as an index for the extent of liver damage.

Furthermore, 50 age- and sex-matched healthy normolipidemic people, referring to the hospital laboratory were selected as our comparison group and their serum lipid profile was measured. Data were analyzed by SPSS. χ2, one-way analysis of variance (ANOVA) and Student’s t test were used. A p value <0.05 was considered statistically significant.

## Results

In our study, the most common causes of cirrhosis were HBV (53%) followed by cryptogenic cirrhosis (18%) (Table 1). The most affected age group was 41 to 50 years so that 90% of cirrhotic patients were over 41 years of age. There was a significant (p=0.03) difference in the frequency of cirrhotic patients among different age groups. Fifty-eight percent of patients were male and 42% were female. According to Child criteria, 11 (22%) patients had score "A," 14 (28%) score "B," and 25 (50%) had score "C." According to MELD criteria, 10 patients had scores <10, 15 had scores between 11 and 18, 17 between 19 and 24, and 8 had scores>25. There was a significant (p<0.05) negative correlation between liver damage-according to MELD and Child criteria-and serum total, HDL and LDL cholesterol level (p<0.05) (Table 2). The more severe the liver damage is, the more decline in lipid levels is detected, especially in LDL and total cholesterol levels. However, no correlation was observed between the serum TG level and the extent of liver damage.

All four variables (HDL, LDL, total cholesterol and TG) were significantly lower in cirrhotic patients than in the comparison group(Table 3).

**Table 1:** Causes of cirrhosis in patients

	**No.**	**%**	**Cumulative frequency (%)**
HBV	27	53	53
HCV	4	8	61
HCB and HCV	2	4	65
Autoimmune	2	4	69
Chronic hepatitis congestion	4	8	76
Wilson's disease	1	2	78
Hemochromatosis	2	4	82
Cryptogenic cirrhosis	9	18	100
Total	51	100	

**Table 2:** Lipid profile in patients with liver damage according to Child and MELD criteria. All data are presented as mean±SD.

	**n**	**LDL (mg/dL)**	**HDL (mg/dL)**	**Total cholesterol (mg/dL0)**	**TG (mg/dL)**
**Child A**	11	107.6±29.5	49.0±21.6	166.5±37.9	121.9±57.7
**Child B**	14	96.4±58.5	40.0±14.3	161.2±71.5	90.6±50.5
**Child C**	25	59.5±28.9	37.4±15.5	121.2±31.7	92.0±48.7
**MELD ≤10**	10	98.3±25.4	51.5±20.6	156.2±34.5	111.5±57.4
**11≤MELD≤18**	15	96.9±62.1	39.0±14.9	162.1±73.4	98.2±44.7
**19≤MELD≤24**	17	69.7±30.8	36.8±16.8	124.7±35.3	96.5±58.1
**MELD ≥25**	8	50.0±24.8	31.8±10.4	104.0±26.0	85.3±48.4

**Table 3:** Mean values of lipid profiles in cirrhotic patients in comparison with the comparison group

**Lipid**	**Cirrhotic patients (mg/dL)**	**Comparison group (mg/dL)**	**p value**
**LDL cholesterol**	80.5	137.2	0.025
**HDL cholesterol**	40.7	44.5	0.043
**Total cholesterol**	138.9	184.6	0.030
**TG**	82.2	187.8	0.011

## Discussion

We found that lower lipid levels are found in patients with liver diseases, and all four studied variables (HDL, LDL, total cholesterol and TG) were significantly lower in cirrhotic patients than in the comparison group. Furthermore, the amount of decrement in the serum HDL, LDL and total cholesterol (but not TG) had a positive correlation with the severity of liver damage.The significant decline in the serum total cholesterol and TG levels in cirrhotic patients compared with healthy people has been confirmed earlier in other studies, which is reasonably expected since liver biosynthesis has been reduced. For instance, the same results were obtained in a study by Mehbob, et al, in 2007, who studied 160 patients with chronic liver diseases. There were significant declines in the serum total cholesterol and TG levels of patients. Another study in Greece was performed by Siagris on 155 patients infected with HCV and 138 healthy people who served as the comparison group, where the serum total cholesterol level was lower in patients than the comparison group. Final results were correlated with total cholesterol and LDL and in cases of genotype 3a, lower levels of TG, HDL and LDL, higher levels of hepatitis C viral load and higher grade of liver steatosis were found comparing to other genotypes. In this study, the final results also showed that liver damage is correlated with total cholesterol, HDL and LDL but not with TG levels. Salimoghlou found that HDL level is lower in Child-Pugh B than Child-Pugh A and apo-A level is the most affected factor in those with liver damage. In this study, the change in HDL level was higher in Child A than B, and higher in Child B than C which shows that that is the severity of liver function that causes HDL level to decline.

Perales found that in patients with chronic liver disease without cholestasis, LDL, HDL and VLDL levels decline and become worse as the disease progresses. This finding is in keeping with our observations that in severe liver disease as the liver function deteriorates, more decline is observed in LDL, HDL and total cholesterol levels. In a study performed by Cicognanic there was an obvious decline in total cholesterol level in patients with chronic liver disease in comparison with controls. In our study, a significant difference was observed between patients and the comparison group in all lipid profile values studied (p<0.05).

It is therefore concluded that the amount of decrements measured in the levels of serum total cholesterol, LDL and HDL in patients with cirrhosis are related to the progress in cirrhosis. Further studies are needed to assess the predictive values of measuring lipid profiles as a mean to estimate the extent of liver damage in cirrhotic patients.
